# Worldwide Genotyping in the Planktonic Foraminifer *Globoconella inflata*: Implications for Life History and Paleoceanography

**DOI:** 10.1371/journal.pone.0026665

**Published:** 2011-10-20

**Authors:** Raphaël Morard, Frédéric Quillévéré, Christophe J. Douady, Colomban de Vargas, Thibault de Garidel-Thoron, Gilles Escarguel

**Affiliations:** 1 CNRS UMR 5276 Laboratoire de Géologie de Lyon: Terre, Planètes, Environnement, Université Lyon 1, Villeurbanne, France; 2 CNRS UMR 7144 Evolution du Plancton et PaléoOceans, Station Biologique de Roscoff, UPMC, Roscoff, France; 3 CNRS UMR 5023 Ecologie des Hydrosystèmes Fluviaux, Université Lyon 1, Villeurbanne, France; 4 Institut Universitaire de France, Paris, France; 5 CEREGE UMR6635, Aix-Marseille Univ, Aix-en-Provence, France; 6 CEREGE UMR6635, CNRS, Aix en Provence, France; Paleontological Institute of Russian Academy of Science, United States of America

## Abstract

The planktonic foraminiferal morpho-species *Globoconella inflata* is widely used as a stratigraphic and paleoceanographic index. While *G. inflata* was until now regarded as a single species, we show that it rather constitutes a complex of two pseudo-cryptic species. Our study is based on *SSU* and *ITS* rDNA sequence analyses and genotyping of 497 individuals collected at 49 oceanic stations covering the worldwide range of the morpho-species. Phylogenetic analyses unveil the presence of two divergent genotypes. Type I inhabits transitional and subtropical waters of both hemispheres, while Type II is restricted to the Antarctic subpolar waters. The two genetic species exhibit a strictly allopatric distribution on each side of the Antarctic Subpolar Front. On the other hand, sediment data show that *G. inflata* was restricted to transitional and subtropical environments since the early Pliocene, and expanded its geographic range to southern subpolar waters ∼700 kyrs ago, during marine isotopic stage 17. This datum may correspond to a peripatric speciation event that led to the partition of an ancestral genotype into two distinct evolutionary units. Biometric measurements performed on individual *G. inflata* from plankton tows north and south of the Antarctic Subpolar Front indicate that Types I and II display slight but significant differences in shell morphology. These morphological differences may allow recognition of the *G. inflata* pseudo-cryptic species back into the fossil record, which in turn may contribute to monitor past movements of the Antarctic Subpolar Front during the middle and late Pleistocene.

## Introduction

Planktonic foraminifera are pelagic protists whose calcareous shells have built up one of the most complete and continuous fossil archive of biodiversity changes over the last 180 Myrs. The biogeography of planktonic foraminiferal morpho-species appears to correlate to hydrographic conditions of latitudinal oceanic provinces [1], [2]. Paleoceanographers derive reconstructions of past climates based on empirical relationships between extant environmental parameters of the surface oceans (e.g., temperature, primary production) and the abundance or chemical composition of shells of individual morpho-species from surface sediment samples [3], [4]. These correlations lie on the working assumption that each species has its own, stable habitat preferences that are transferable back into the past to reconstruct changes of water masses physical properties. The accurate recognition of individual species is therefore mandatory for the use of planktonic foraminifera as paleoceanographic proxies.

Extant species of planktonic foraminifera have been defined based on diagnostic characters of their shell (the so-called *morpho-species* concept), primarily described from fossil specimens extracted from sediments [5], [6]. Yet, molecular analyses applied to living specimens have challenged this concept by demonstrating that this classical taxonomy underestimates planktonic foraminiferal diversity. Each morpho-species analyzed so far actually comprises up to seven distinct genotypes [7], most of which exhibiting a distinct biogeography and/or ecology [8]–[10], and even sometimes subtle but statistically significant differences in shell morphology [11]–[14]. Although strictly identical ribosomal DNA (rDNA) genotypes range across huge geographic distances, different genotypes within a single morpho-species generally display peculiar distributions related to water masses properties [8], [10], [13], [14]. Together with molecular clock analyses calibrated on the fossil record [9]–[11], these results strongly support the hypothesis that distinct genotypes within the classical morpho-species actually correspond to cryptic, or rather pseudo-cryptic biological species, i. e., sibling species that can be differentiated based on subtle morphological features [15]. In this study, we investigate the rDNA genetic diversity and biogeography within the morpho-species *Globoconella inflata* d'Orbigny 1839.

*Globoconella inflata* is a macro-perforate, non-spinose morpho-species of planktonic foraminifera (Globorotalioidea), which originated 4.14 Ma ago in transitional waters of the Southwest Pacific [16], and which spread 2.09 Ma ago into the world oceans [17]. Today, this thermocline-dweller species [18], [19] is particularly abundant in transitional and subtropical waters of both hemispheres [1].

**Figure 1:**
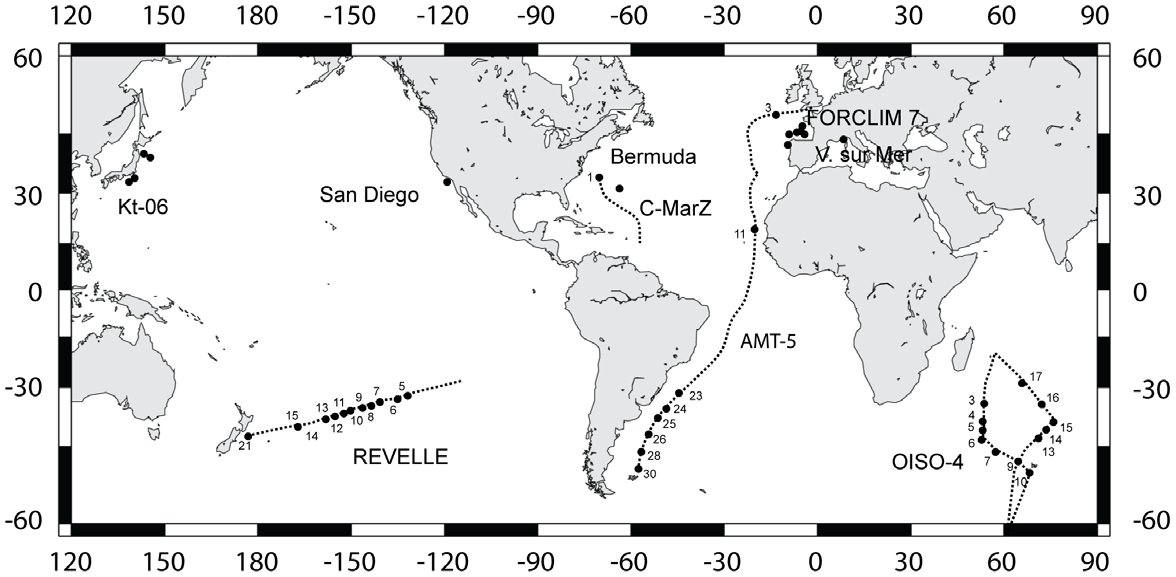
Sample map. Geographic location and labels of the oceanic stations sampled during the cruises FORCLIM 7, AMT-5, C-MarZ, OISO-4, REVELLE, KT-06-11 and offshore Villefranche-sur-mer, San Diego and Bermuda. Dashed lines represent ship routes and black circles are collecting stations from where *Globoconella inflata* specimens have been genetically analyzed.

Our study focuses on the genetic variations in *Small SubUnit* (*SSU*) and *Internal Transcribed Spacer* (*ITS*) rDNA sequences of specimens collected worldwide from plankton tows (Figure 1). The *SSU* rDNA sequences have been used in most studies dealing with foraminiferal genetic diversity [7], [20], [21]. The *ITS* rDNA sequences evolve significantly faster than *SSU* rDNA and can be used to discriminate recent events of pseudo-cryptic speciation [10]. In this study, we document the occurrence of two distinct genotypes of *Globoconella inflata*, supported by both *SSU* and *ITS* analyses. These genotypes display specific, strictly allopatric biogeographic distributions on a global scale, as well as statistically significant differences in shell morphology. Ultimately, morpho-genetic delineation of both pseudo-cryptic species of *G. inflata* may be used to improve paleoceoanographic reconstructions, especially the glacial-interglacial latitudinal swings of the Antarctic Subpolar Front since the mid-Pleistocene transition.

## Results

### Genetic Variation in *Globoconella inflata*

*SSU* rDNA sequences of 21 *Globoconella inflata* individuals randomly selected from contrasted water masses (Table 1, Figure 1) reveal small but robust molecular differences between specimens collected north and south of the Antarctic Subpolar Front (Table 2, Figure S1). This pattern of genetic diversity is confirmed based on the analysis of the complete *ITS* array (*ITS-1*, *5.8S* and *ITS-2*) of 80 specimens from 41 stations that cover the entire environmental range of the morpho-species. In order to assess the intra-individual level of genetic variations, clones from single cells were also characterized in both *SSU* (33) and *ITS* (55) rDNA (Table 3). All together, our dataset includes 50 *SSU* rDNA sequences and 135 *ITS* rDNA sequences from 83 individuals. The remaining 414 individuals were genetically characterized through Restriction Fragment Length Polymorphism analysis (RFLP).

**Table 1:** Location of the sampling stations, with indications of the number of sequenced and genotyped specimens of *Globoconella inflata* (in parenthesis, number of copies from the same individual).

Cruise	Station	Latitude	Longitude	Environmental data	Number of sequenced specimens (SSU)	Number of sequenced specimens (ITS)	Number of RFLP identifications	Genotype
C-MarZ	1	33°54′N	69°93′W	CTD (T)	2	5 (3; 16)	79	I
OISO-4	3	35°00′S	53°30′E	CTD (T, F)	2	1 (17)	3	I
OISO-4	4	40°01′S	52°53′E	CTD (T, F)	2 (13)	3	8	I
OISO-4	5	42°31′S	52°29′E	CTD (T, F)		1	7	I
OISO-4	6	45°00′S	52°05′E	CTD (T, F)	4	5 (19)	0	II
OISO-4	7	47°40′S	58°00E	CTD (T, F)		8	8	II
OISO-4	9	48°31′S	64°59′E	CTD (T, F)	3 (2)	2	0	II
OISO-4	10	50°40′S	68°24′E	CTD (T, F)		0	2	II
OISO-4	13	44°58′S	73°21′E	CTD (T, F)		2	8	I
OISO-4	14	42°30′S	74°53′E	CTD (T, F)		2	18	I
OISO-4	15	40°00′S	76°24′E	CTD (T, F)		3	13	I
OISO-4	16	34°59′E	73°28′E	CTD (T, F)		0	3	I
OISO-4	17	29°59′S	66°24′E	CTD (T, F)		1	1	I
FORCLIM 7	I	42°37′N	10°02′W	CTD (T, F)		0	15	I
FORCLIM 7	II	44°20′N	8°45′W	CTD (T, F)		1	9	I
FORCLIM 7	III	45°35′N	7°33′W	CTD (T, F)	1 (2)	1	9	I
FORCLIM 7	V	45°37′N	7°33′W	CTD (T, F)		0	3	I
FORCLIM 7	VIII	45°38′N	7°36′W	CTD (T, F)		1	4	I
FORCLIM 7	XV	45°06′N	5°38′W	CTD (T, F)		3	17	I
FORCLIM 7	XXII	45°56′N	6°15′W	CTD (T, F)		0	50	I
FORCLIM 7	XXVII	46°36′N	5°49′W	CTD (T, F)		0	17	I
KT-06	C	39°00′N	145°00′E	CTD (T,F)	1	2	32	I
KT-06	E	34°00′N	140°00′E	CTD (T,F)	1	1	1	I
KT-06	F	33°00N	139°00E	CTD (T,F)		3	9	I
KT-06	G	33°00N	139°00E	CTD (T,F)		0	1	I
REVELLE	5	31°35′S	127°83′W	SST		1	3	I
REVELLE	6	32°04′S	130°98′W	SST		1	2	I
REVELLE	7	33°39′S	137°12′W	CTD (T,F)		0	14	I
REVELLE	8	34°03′S	140°05′W	SST		1	6	I
REVELLE	9	34°73′S	143°27′W	CTD (T,F)	1	1	2	I
REVELLE	10	35°39′S	146°29′W	SST	1	1 (3)	1	I
REVELLE	11	36°07′S	149°48′W	CTD (T,F)		1	8	I
REVELLE	12	36°73′S	152°58′W	SST		1	5	I
REVELLE	13	37°45′S	156°00′W	CTD (T,F)		1	6	I
REVELLE	15	39°62′S	166°69′W	CTD (T,F)		1	5	I
REVELLE	21	42°95′S	176°26′E	SST		1	45	I
Villefranche-sur-Mer	N/A	43°40′N	07°15′E	N/A		1 (2)	0	I
San Diego	N/A	33°16′N	118°08′E	N/A		1 (2)	0	I
Bermuda	1	32°08′N	64°33′W	N/A		1	0	I
Bermuda	2	32°20′N	64°33′W	N/A		1	0	I
Bermuda	3	32°20′N	64°33′W	N/A		1	0	I
AMT-5	3	47°98′N	13°20′W	CTD (T,F)		2	0	I
AMT-5	11	19°71′N	20°50′W	CTD (T,F)		1	0	I
AMT-5	23	31°37′S	44°52′W	CTD (T,F)		3	0	I
AMT-5	24	35°29′S	48°52′W	CTD (T,F)		2	0	I
AMT-5	25	38°50′S	51°55′W	CTD (T,F)	1	3	0	I
AMT-5	26	42°14′S	54°27′W	CTD (T,F)		3(2)	0	I
AMT-5	28	46°03′S	56°42′W	CTD (T,F)	2 (8; 9)	4	0	II
AMT-5	30	49°79′S	57°62′W	CTD (T,F)		1	0	II
				Total	21 (50)	80 (135)	414	

**Table 2:** Inter-individual patristic distances (substitutions per site) measured on phylogenetic trees obtained from the five datasets analyzed (see Methods for details about the co nstruction of the datasets).

		SSU		CleITS		CloITS		LarITS		ComITS	
		Min	Max	Min	Max	Min	Max	Min	Max	Min	Max
Type I	Median	4.77E-03		3.84E-02		1.01E-02		1.56E-02		1.37E-02	
	95% CI	9.25E-08	1.23E-02	1.41E-02	8.21E-02	3.30E-03	3.76E-02	2.60E-03	3.52E-02	2.00E-03	3.18E-02
	Min-Max	2.00E-10	1.44E-02	1.46E-07	1.02E-01	2.00E-10	5.55E-02	2.00E-10	5.68E-02	2.00E-10	4.32E-02
Type II	Median	7.67E-03		5.24E-02		1.17E-02		1.83E-02		1.37E-02	
	95% CI	2.51E-07	1.42E-02	1.90E-02	9.06E-02	8.40E-07	2.82E-02	2.60E-03	4.33E-02	8.10E-07	3.21E-02
	Min-Max	7.25E-08	1.65E-02	9.60E-03	9.59E-02	2.00E-10	3.54E-02	2.00E-10	5.91E-02	2.00E-10	4.44E-02
Type I vs. Type II	Median		1.38E-01		4.37E-02		6.70E-02		4.83E-02		
	95% CI	1.34E-02	3.13E-02	1.03E-01	1.80E-01	3.10E-02	5.72E-02	4.88E-02	8.86E-02	3.54E-02	6.75E-02
	Min-Max	1.03E-02	3.47E-02	8.26E-02	2.11E-01	5.00E-03	6.90E-02	3.82E-02	1.13E-01	2.87E-02	8.54E-02

The median, 95% non-parametric confidence interval, and minimum and maximum patristic distance values are given within and among genotypes.

**Table 3:** Intra-individual patristic distances (substitutions per site) measured on phylogenetic trees obtained from four of the five analyzed datasets (no available distances for the CleITS dataset; see Methods for details about the construction of the datasets).

		SSU		CloITS		LarITS		ComITS	
		Min	Max	Min	Max	Min	Max	Min	Max
Oi 375	Median	6.39E-03							
	95% CI	2.00E-10	1.27E-02						
	Min-Max	2.00E-10	1.43E-02						
AM605	Median	6.51E-03							
	95% CI	7.25E-08	1.12E-02						
	Min-Max	7.80E-08	1.12E-02						
AM609	Median	9.07E-03							
	95% CI	8.25E-08	1.33E-02						
	Min-Max	8.24E-08	1.34E-02						
CM115	Median			6.67E-03		1.03E-02		7.79E-03	
	95% CI			4.15E-07	1.50E-02	3.98E-10	2.34E-02	2.16E-07	1.56E-02
	Min-Max			2.00E-10	1.50E-02	2.00E-10	2.60E-02	2.00E-10	1.77E-02
Oi265	Median			1.16E-02		1.30E-02		1.17E-02	
	95% CI			1.04E-07	1.95E-02	1.41E-07	2.11E-02	3.33E-07	1.78E-02
	Min-Max			2.00E-10	2.18E-02	2.00E-10	2.12E-02	7.06E-08	1.79E-02
Oi689	Median			5.01E-03		7.74E-03		5.83E-03	
	95% CI			1.99E-07	1.03E-02	1.92E-07	1.82E-02	2.05E-07	1.37E-02
	Min-Max			2.00E-10	1.17E-02	2.00E-10	2.08E-02	2.00E-10	1.56E-02
Re1010	Median							4.94E-03	
	95% CI							1.09E-03	5.79E-03
	Min-Max							8.90E-04	5.83E-03

The median, 95% non-parametric confidence interval, and minimum and maximum patristic distance values are given within 7 cloned individuals. Specimen Re-1010 displays a Type I-like sequence with a Type II RFLP pattern.

**Figure 2:**
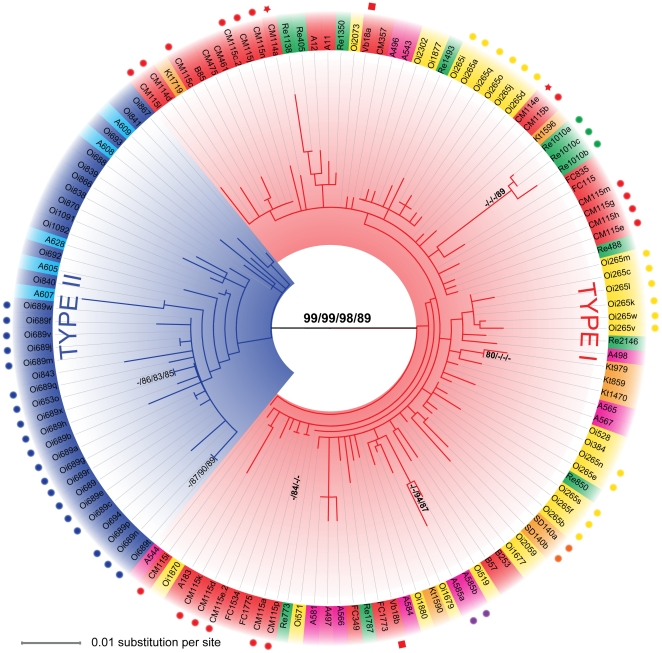
Phylogenetic analysis of *Globoconella inflata*. *ITS*-based evolutionary relationships between 135 clones of *G. inflata* from 41 localities in the world oceans (see Table 1 and Figure 1 for station names and locations). This Maximum Likelihood inference shows the relationships between the two phylotypes (Type I in red and Type II in blue). The bootstrap scores (500 replicates) greater than 80% are given next to branches for each dataset following a *CleITS*/*CloITS*/*LarITS*/*ComITS*-dataset order. The scale and branch lengths are given in % of nucleotide substitution per site. The colors associated to leaf labels indicate geographic area of collection: blue = subpolar Indian Ocean; light blue = subpolar South Atlantic; Pink = South Atlantic north of the Subpolar Front; Yellow = Indian Ocean north of the Subpolar Front; red = North Atlantic; green = South Pacific; Orange = North Pacific. Circles, stars and squares associated to specific colors indicate clones sequenced from the same individuals.

*SSU* and *ITS* rDNA sequences of *Globoconella inflata* cluster into two clearly distinct groups called here Type I and Type II (Figure 2 and Figure S1). In datasets analyzed (1 *SSU* and 4 *ITS*; see Material and Methods), patristic distances between the two genotypes are markedly higher than those measured within each genotype (Table 2). Moreover, genetic variations within individuals are equal or greater than differentiations amongst populations from the three oceanic basins, making these sequences uninformative at the population level (Table 3). The node separating Types I and II of *G. inflata* is supported by bootstrap values ≥98% (except for the *comITS* dataset, 89%), whereas no branch support higher than 80% is observed within phylotypes, with only few exceptions for small clusters of terminal branches (Figure 2).

**Figure 3:**
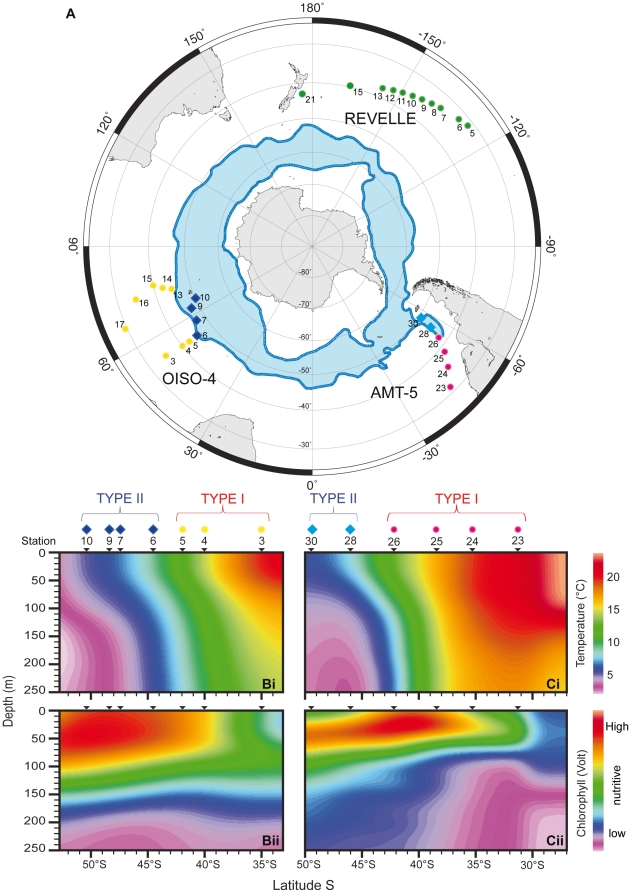
Geographical and ecological distribution of genotypes. Latitudinal distribution of Types I and II of *Globoconella inflata* in the South Hemisphere (the North Hemisphere contains representatives of Type I only). (A) Polar projection of the cruises AMT-5 (September-October 1997), OISO-4 (January–February 2000) and REVELLE (January–february 2004); the position of the Antarctic Circumpolar Current is shown in blue [61]. Temperature and fluorescence profiles (0–250 m) are given for the cruise OISO-4 (B*i* and B*ii*) and AMT-5 (C*i* and C*ii*); occurrences of genotypes (circles for Type I; diamonds for Type II) are given for each station, positioned with latitudes. Colors as in Figure 1.

A unique specimen (Re-1010) collected in the subtropical south Pacific during the cruise *REVELLE* (Figure 1, 3), displays an atypical Type I *ITS* rDNA sequence but a Type II RFLP pattern. Based on the *CleITS* alignment (652 unambiguously aligned sites), sequences obtained from this specimen (3 clones were characterized from two distinct PCR, showing observed pairwise distances between 0.006676 and 0.002387 substitution per site) share 13 synapomorphous sites with Type I, 4 with Type II (all found in a 28 bp-long region), and possess 5 autapomorphous sites when compared to the 60% consensus sequences of Types I and II as inferred by SEAVIEW [22]. Even if rather ambiguous in the studied dataset, Indel locations do seem to roughly follow the same pattern. Despite its RFLP pattern, this individual clearly clusters within Type I in *ITS* phylogeny, although being slightly divergent and decreasing the bootstrap support between the two genotypes (Figure 2). Moreover, its *SSU* rDNA sequence clearly belongs to Type I. Since we found no clear evidence of hybridization but could not reject it based on the available data, we currently favor the hypothesis that this isolated specimen coming from a well-sampled oceanic area (Table 1) could be the representative of a distinct Type I-population, which remains underrepresented in our dataset.

### Geographic Distribution of *Globoconella inflata* Genotypes

Our molecular dataset, including both sequence and RFLP analyses, originates from 49 sampled stations that cover the entire biogeographic/environmental spectrum known for *Globoconella inflata*, except for northern high-latitudes (see next section). Type I and Type II exhibit a strictly allopatric distribution. Type I is found within the subtropical and transitional water masses of the world oceans, whereas Type II is restricted to the cold, vertically mixed and nutrient-rich water masses of the Subpolar Southern Ocean (Figure 3A). This distribution pattern is apparently not primarily controlled by water productivity. Indeed, surface waters with the highest fluorescence values matching maximum chlorophyll concentrations yield different genotypes in the South Atlantic (Type I in stations 25 and 26 of AMT-5; Figure 3C) and South Indian Ocean (Type II in stations 7, 9 and 10 of OISO-4; Figure 3B). However, in both Atlantic and Indian Ocean basins, the biogeographic boundary between the two genotypes corresponds to the location of the North Subpolar Front, where Sea Surface Temperatures (SSTs) range between 8°C and 12°C (Figure 3B, C).

### Bipolar Distribution of *Globoconella inflata*

**Figure 4:**
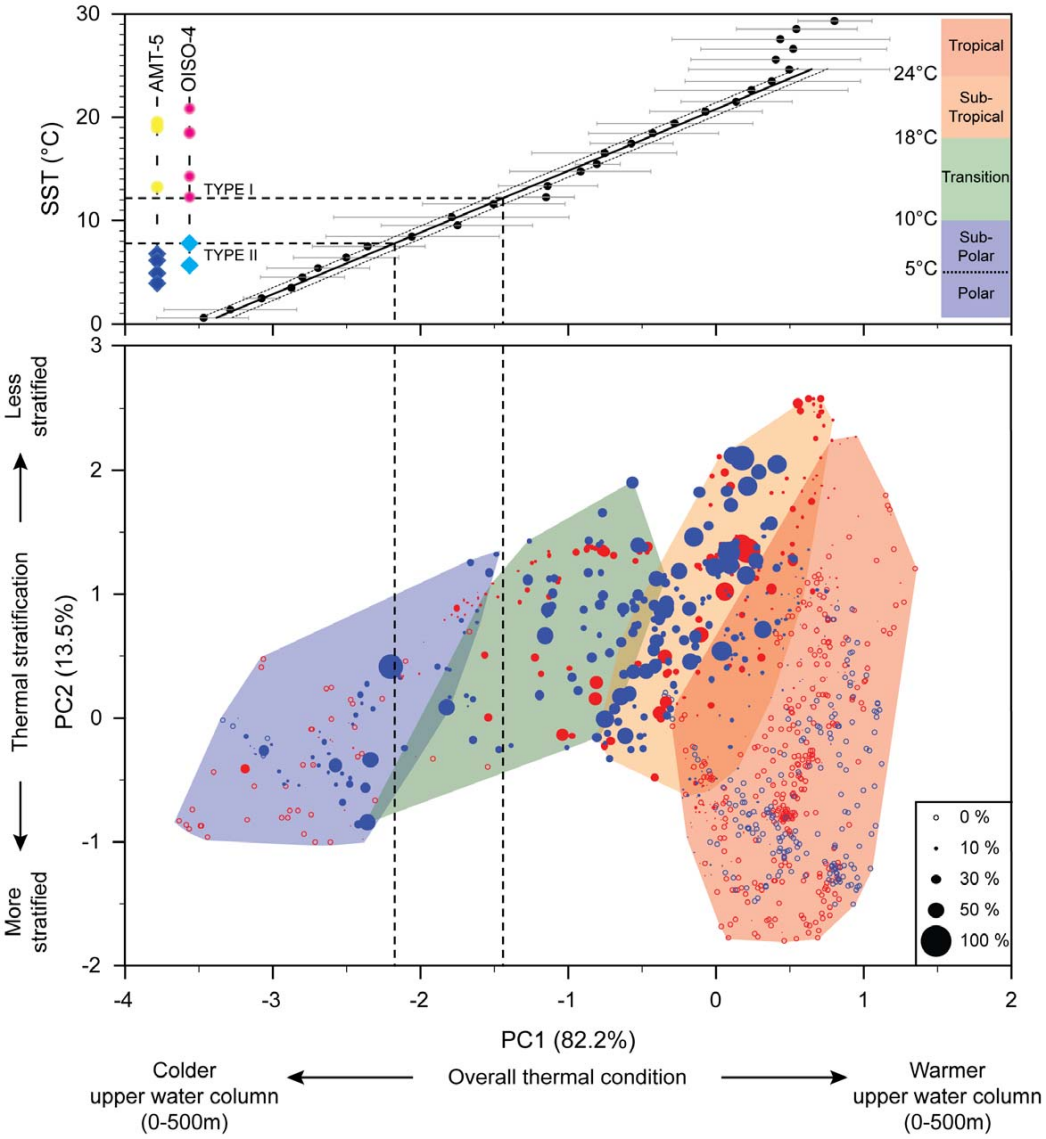
Morpho-species *vs.* genotype distributions. The bubble biplot allows the direct comparison of the relative abundance of *Globoconella inflata* (proportional to filled circle size; empty circle: absent) in surface sediments of 1265 localities from the North (in red) and South (in blue) Hemisphere with the thermal structure of the 500 upper meters of the water column. A least square regression between the mean values of PC1 for each 1 SST-°C interval and SST (Sea Surface Temperature = 10 meters temperature following [3]; standard deviation indicated by a gray line) shows the good relationship between these two descriptors in the 0–25°C-SST interval and illustrates the clear thermal boundary (between 8 and 12°C based on the studied samples) between the two genotypes for the cruises AMT-5 and OISO-4 (colors and symbols as in Figure 2).

Given the lack of plankton tow samples from northern high latitudes in our dataset, we cannot check whether the subpolar waters of the Northern Hemisphere host the Type II of *Globoconella inflata*, as it would be expected in a bipolar biogeographic pattern [23], [24], or, alternatively, another genotype. In order to circumvent this sampling limitation, we performed a biogeo-hydrographic analysis of the overall distribution of *G. inflata* in surface sediments samples from the *Brown University Foraminiferal Database* [25]. The BFD records the absolute abundances of 37 extant morpho-species of planktonic foraminifera at 1265 core-top sites of the global oceans. For each BFD site, thirteen mean annual temperatures measured between sea surface and 500 m depth, i.e., the potential depth-habitat range of *G. inflata*, were extracted from the World Ocean Atlas [26]. A principal component analysis was performed on these 13 temperatures values×1265 localities allowing the direct comparison between the synthetic descriptors of the thermal structure of the water column (the resulting principal components) and the abundance of *G. inflata* (Figure 4). The resulting PC1 is a mean thermal state of the upper 500 m of the water column; PC2 contrasts the mean annual temperatures of the first 125 m (negative weight) with the temperatures recorded between 150 and 500 m (positive weight; Figure S2). Excepted a very few (11) core-top samples located in the vicinity of the Gulf Stream [27] where *G. inflata* is sparsely recorded, the morpho-species is absent in northern subpolar waters at thermal conditions where Type II occurs in southern high latitudes. The latitudes at which *G. inflata* disappears in the Northern Hemisphere exhibit mean SST values identical to those measured for the biogeographic boundary between the two genotypes in the Southern Hemisphere (Figure 4). Although we acknowledge that further samples from the Northern Hemisphere (Figure 1) would be helpful to definitely exclude the presence of another genotype in this area, our observations clearly support an absence of a specific or shared arctic subpolar genotype.

### Biometry

**Figure 5:**
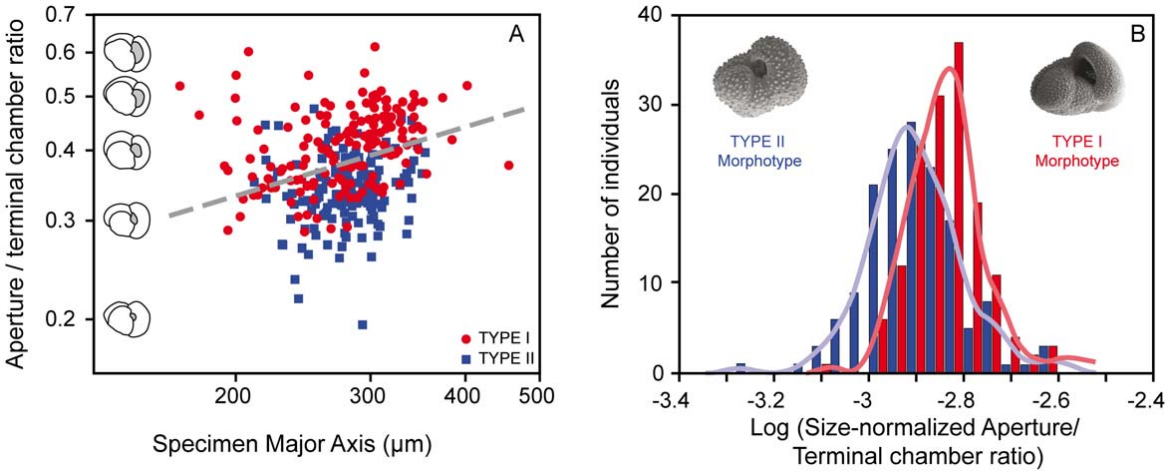
Morphological differences between *Globoconella inflata* genotypes. **A**. Log-Log Biplot of specimen major axis *vs.* aperture/terminal chamber length ratio for 306 specimens collected during the cruise AMT-5 in the South Atlantic. All specimens collected north of the Subpolar Front are considered to be representatives of Type I; all others are considered as Type II. The discriminant boundary that maximizes the separation between the two genotypes is represented by a gray dashed line. **B**. Histograms and Gaussian kernel densities of the log-ratio between the aperture/terminal chamber length ratio and the specimen major axis.

A biometric analysis was performed on 306 non-genetically characterized *Globoconella inflata* specimens from plankton tows across the Antarctic subpolar frontal system (AMT-5 cruise). Rather than direct morpho-genetic comparisons, we favored this approach because of the highly unbalanced molecular sampling between Types I and II. Based on the procedure by [28] and the available genetic dataset, the probability to get a mix of Type I and Type II specimens in a given locality north or south of the Subpolar Front is <0.035% at the 95% confidence level. This clearly indicates that non-genotyped individuals currently living, and thus collected north (n = 154) and south (n = 152) of the front are very likely to be representatives of Type I and Type II, respectively. Measurements of overall shell size and apertural relative size descriptors reveal a weak, but highly significant difference among the two sets of individuals (genotypes) sampled on each side of the front (Figure 5). A discriminant analysis involving both populations indicates a highly significant differentiation among genotypes (Wilk's λ = 0.858; F = 25.06; df = 2, 303; p = 8.48×10^−11^), which is also non-parametrically evidenced based on the log-ratio between the aperture/terminal chamber ratio (a size-normalized apertural length) and the specimen major axis (Figure 5B; equal-median Mann-Whitney test: U = 6607, p = 4.5×10^−11^; same-distribution Kolmogorov-Smirnov test: D = 0.358, p = 3.4×10^−9^). At an identical overall shell size, specimens collected north of the front display a significantly higher size-normalized apertural length than those collected south of the front. This morphological distinction of two varieties of *G. inflata* agrees with surface sediment data by [29], which differentiated, in the SW Pacific, a low to mid latitudes morphotype and a subpolar morphotype, this later being characterized by a smaller umbilico-ventral aperture. Even if our simple morphometric descriptor does not yet allow a powerful discrimination between the two genotypes (the resulting discriminant function allows the correct genotype classification of only 66% of the analyzed specimens), our results suggest that further, more sophisticated morphometric analyses will probably make possible to morphologically characterize the two genotypes in fossil assemblages.

## Discussion

### Ribosomal DNA for Identifying Genetic Variability in Planktonic Foraminifera

Ribosomal DNA provides useful markers for phylogenetic systematics in protist taxa, especially at the species level [30]–[35]. However, *ITS* rDNA-based phylogenetic reconstructions have to be interpreted cautiously because intragenomic variations can lead to overestimates of the diversity or to the incorrect finding of cryptic species, as shown for example for the dinoflagellate cluster *Symbiodinium* spp. [36]. In the case of the planktonic foraminifera *Globoconella inflata*, the branch separating the two phylogroups we evidence is highly supported by both *SSU* and *ITS* analyses. In addition, no clone from individual assigned to Type II clustered within the Type I, and inversely. These two lines of evidence strongly suggest that the two phylogroups evolved as separated evolutionary units that split off during the past.

The biology of planktonic foraminifera remains poorly understood, especially because none was able yet to obtain complete life cycles of these organisms in laboratory cultures. It is therefore not possible to conduct mating experiments to test those genotypes of *G. inflata* for the Mayr's biological species concept (groups of interbreeding natural populations that are reproductively isolated from other such groups). Consequently, the appropriate way to prove the reliability of the rDNA-defined phylogroups as different biological species is to combine evidences from different life history traits that directly or indirectly relate to the interbreeding criterion [37]. The highly supported genetic characterization, the complete geographical and ecological disruption, and the morphological differences evidenced among genotypes of *G. inflata* (Figure 3, 4, 5) argue together for a biological species-level differentiation among Types I and II.

Abundant sediment data make *G. inflata* a well known planktonic foraminiferal morpho-species in the fossil record, both in terms of origination and paleogeographic distribution [38], [16]. It initially appeared during the early Pliocene (4.14 Ma; [16]) in the transitional Southwest Pacific before definitely invading the transitional and subtropical waters of both hemispheres 2.09 myrs ago [17]. Restricted to these environments during the first million of years of its evolution, *G. inflata* expanded its geographic range to the southern subpolar waters at ∼700 ka [39], [40]. Previous studies interpreted this event as an intra-specific migration associated with an adaptation to cold waters of the high latitudes. Such interpretation implied that the morpho-species exhibits a wider temperature tolerance in the southern than in the northern Hemisphere, where its distribution does not range as far north as the transitional waters. Alternatively, our data strongly suggest that this invading datum may correspond to a peripatric [41] speciation in which an ancestral genotype evolved into two evolutionary significant units.

### Cryptic Diversity as a Tool for Monitoring Past Migrations of the Antarctic Subpolar Front

In planktonic foraminifera, co-occurrences of distinct genotypes of the same morpho-species may induce significant bias in paleoceanographic reconstructions [3], [8], [10], [13], [28], [42]–[46]. Consequently to its evolutionary history, *Globoconella inflata* falls in a unique case because no co-occurrence of cryptic species has been observed to date, in an almost global dataset. As a consequence, the cryptic diversity in *G. inflata* does not affect paleoceanographic reconstructions as soon as they rely on calibrations that have been completed based on modern specimens collected within the biogeographic range of a single genotype. For example, most stable isotope studies focusing on the ecology of current *G. inflata* are based on the analysis of specimens that have been collected within the geographic range of Type I [19], [47]–[51]. These studies are consequently not affected by the existence of two genotypes with different ecologies. Stable isotope analyses (and their paleoceanographic inferences) based on current specimens collected through the Antarctic Subpolar Front [52]–[53], should however be interpreted cautiously, because the observed isotopic signals may be biased by the way each of the two genotypes fractionate oxygen and/or carbon isotopes. Furthermore, the transfer functions used by paleoceanographers to predict past sea surface temperatures are calibrated based on abundances of current planktonic foraminiferal species in surface sediments. Most of these calibrations being basin-wide based, they mix data originating from the ranges of Type I and Type II of *G. inflata* in the South Hemisphere, then potentially affecting the resolving power of the transfer functions [13].

On the other hand, cryptic diversity in *Globoconella inflata* may be a powerful tool to monitor past migrations of the Antarctic Subpolar Front during the glacial and interglacial stages of the middle and late Pleistocene. Though biometric studies of *G. inflata* specimens from surface sediments are needed to test our hypothesis, several arguments argue for a direct recognition of Type I and Type II back into the middle and late Pleistocene. First, only two extant cryptic species have been described to date, making feasible to transfer the genetic information to the interpretation of the fossil record. Second, since the Type II of *G. inflata* has permanently inhabited the subantarctic waters since ∼700 ka, Types I and II have probably maintained their structural sensitivity to temperature conditions and fluctuations. Third, our rough preliminary biometrical analysis clearly suggests that the genotype of shell samples could be statistically inferred, e.g., based on a geometric morphometry analysis of the apertural/final chamber relationship. Based on the two first points, application of this morphometric approach should work equally in the fossil record, making the identification of cryptic species in *G. inflata* a useful paleontological proxy. Such identification should therefore be considered as a promising tool to track the past glacial/interglacial oscillations of the Antarctic Subpolar Front over the last ∼700 ka, a critical parameter for global paleoceanographic and paleoclimatic reconstructions [54], [55].

## Materials and Methods

### Sample Collection

Living *Globoconella inflata* were collected with plankton tows (64 µm or 100 µm mesh sizes) from the world oceans (Figure 1): (*i*) in the Atlantic Ocean during the cruises AMT-5 (Sept–Oct 1997), C-MarZ (April 2006), FORCLIM 7 (April 2009) and offshore Bermuda; (*ii*) in the Pacific Ocean during the cruises REVELLE (January–February 2004), KT-06-11 (June 2007), and offshore San Diego; (*iii*) in the Indian Ocean during the cruise OISO-4 (January–February 2000). Additional material was collected offshore Villefranche-sur-mer (France) in the Mediterranean Sea. No specific permits were required for the described field studies. The specimens were individually cleaned with a fine brush, isolated on the day of collection into a DNA extraction buffer (see below), and stored at −20°C. In total, we genetically analyzed 497 specimens from 49 open oceanic stations. At most sampling sites, temperature, salinity, and chlorophyll-a fluorescence vertical profiles down to 250 m were obtained by CTD casts.

### DNA Extraction, Amplification and Sequencing

DNA extractions of 497 specimens were performed using the GITC [20] and GITC* [13] extraction buffers. Molecular analyses were carried out using the conserved 18S *SSU* and the more variable *ITS* (*ITS-1*, *5.8S*, *ITS-2*) rDNA sequences. For 21 specimens originating from water masses of contrasted properties (Table 1), we amplified a ∼620 pb fragment localized at the 3′ end of the *SSU* rDNA based on PCR using the foraminiferal specific primers S15rf (5′ GTG CAT GGC CGT TCT TAG TTC 3′) - S19f (5′ CCC GTA CRA GGC ATT CCT AG 3′). For 80 specimens, we further amplified the whole *ITS* enclosing *ITS-1*, *5.8S*, and *ITS-2* (∼1000 pb) using the primers S30f (5′ AAGAGAAGTCGTAACAAGGC 3′) - L5f (5′ TCGCCGTTACTAAGGRAATC 3′). All PCR products were cloned using the TOPO TA cloning kit (Invitrogen). The 50 clones for the *SSU* and 135 clones for the *ITS* were sequenced using an ABI prism sequencer (Applied Biosystem) at the Station Biologique de Roscoff. The new sequences obtained in this study were deposited in Genbank with accession numbers JN164368 to JN164502 and JN164503 to JN164552, for the *ITS* and *SSU* regions, respectively.

### Datasets, Alignment and DNA Sequence Analysis

The 50 *SSU* rDNA sequences obtained were manually aligned using SEAVIEW 4.0 [22]. Best fitted model of evolution was selected by jModeltest v 0.1.1 [56]. Using the selected model of substitution (HKY+G), four discrete categories for the gamma distributions and NNI+SPR tree improvements, a Maximum Likelihood approach implemented in PhyML software [57] with non-parametric bootstrapping (500 pseudo-replicates) was used to assess the most-likely tree topologies.

For the *ITS* array, four steps of alignments were successively used in our phylogenetic analysis. A first alignment (*cleITS*) included only the strictly unambiguous sequences from 54 specimens. In a second alignment (*cloITS*), clones from single specimens were added to test the strength of non-concerted evolution in *Globoconella inflata ITS* rDNA. Sequences that included a few ambiguous sites were added in a third alignment (*larITS*) in order to increase the geographic coverage of the dataset. Finally, three sequences cloned from an atypical individual displaying a Type II RFLP pattern within a Type I population (Re-1010, from REVELLE station 10) were included into a fourth dataset (*comITS*). Each dataset was aligned both manually and automatically using the MUSCLE software implemented in SEAVIEW 4.0 [22]. For both alignments of each dataset, poorly (highly variable) aligned regions were removed from the final alignments using GBLOCKS v. 0.91b [58] with the options allowing “smaller final blocks”, “gap positions within the final blocks” and “less strict flancking positions”. Finally, a consensus of both the manual and automatic alignments after GBLOCKS treatment was built, and the ambiguous positions between both methods were removed from the final alignment. We then used jModeltest v 0.1.1 [56] to select the most appropriate nucleotide substitution models. The (HKY+I+G) model was selected under the Akaike information criterion for the *CleITS* and *LarITS* datasets, while the (TrN+G) and the (HKY+G) were favored for the *CloITS* and the *ComITS* datasets, respectively. Using these models of substitution, four discrete categories for the gamma distributions and NNI+SPR tree improvements, a Maximum Likelihood approach [57] with non-parametric bootstrapping (500 pseudo-replicates) was used to assess the most-likely trees topologies.

### RFLP Analysis

We developed a RFLP protocol based on the *ITS* region to rapidly recognize the genotypes of the 417 *Globoconella inflata* specimens that were not sequenced. After single-cell PCR, the products were digested with the endonuclease *BstNI* (New England Biolabs), which cuts at the sequence CC/WGG, according to the following protocol: 5 µl of the *ITS* rDNA PCR product were directly digested for 2 h at 60°C in a total volume of 10 µl containing 0.1 µl of the enzyme (1 U), 1 µl of the 10× buffer (New England Biolabs), and 3.9 µl of distilled water. Distinct patterns for each genotype were UV-detected after migration of the digested PCR products on 1.5% agarose gel, and ethidium bromide staining. The Type I typically produces a two-band pattern at 400 bp and 600 pb, and sometimes a third band occurs when specimens display length polymorphism between *ITS* copies. The Type II is not cut.

### Biogeography of *Globoconella inflata* from Surface Sediments

Based on the BFD [25], we analyzed the global, environmental distribution of *Globoconella inflata*. The BFD records the absolute abundances of ∼551,600 intact individuals distributed within 37 extant morpho-species over 1265 sample localities from the world surface sediments (median sample size: 379 individuals; 95% Confidence Interval: 235–1033). *Globoconella inflata* is recorded in 566 (44.7%) localities, where it represents an average of 11.4% of the total assemblage (95% C.I.: 0.2%–54.7%).

For each of the 1265 core-tops, we extracted temperature data (annual mean) from the World Ocean Atlas 2005 [26] at the following water depths: 0, 10, 20, 30, 50, 75, 100, 125, 150, 250, 300, 400 and 500 m. In 3.27% (561 over 17,710) of the sample locality×water depth couples analyzed, temperature values were not directly available in the WOA for the 1°^2^ target-cell where the BFD sample station is located. In those cases, we interpolated the missing values from the ≤8 1°^2^ cells immediately surrounding the target-cell (forming a 3°×3° surface area). Interpolated values are weighted averages (weighting factor: inverse angular distance to the target-cell).

Based on this directly available and interpolated mean annual temperature dataset, the synthetic descriptors of the thermal structure of the water column for each of the 1265 sample localities were estimated through a correlation matrix-based Principal Component Analysis of the 13 temperature variables. A bubble biplot of the two first components, representing 95.7% of the overall annual 3D thermal variability, illustrates the distributions of the morpho-species abundances and genotypes occurrences throughout the main climatic provinces of the world oceans (Figure 4).

### Biometrics

Looking at potential morphological differences between the two genotypes of *Globoconella inflata*, we conducted biometric measurements on 306 specimens collected during the cruise AMT-5 across the subpolar frontal zone. Specimens collected north (n = 154) and south (n = 152) of the Antarctic Subpolar Front were assumed to be representatives of Type I and Type II, respectively. The robustness of such an assumption was estimated based on the procedure by [28], which computes the probability that the observed distribution is biased by overlooking rare specimens of the presumably absent genotype. We used the equations:
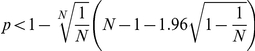


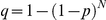
Where *p* is the relative abundance of such a rare genotype, *q* the probability of not having collected a rare genotype among the sampled material, and *N* the total number of collected individuals.

All specimens were mounted on glass cover slips with double side tape and similarly oriented on the umbilical and edge views of their shells, and then digitized under the microscope using an optical image analyzer (OPTIMAS v. 6.51). Length of the major axis of each specimen in edge view (a simple and robust estimator of individual size), together with lengths of the major axes of the terminal chamber and aperture were extracted from digitized outlines. The ratio between the lengths of major axes of the aperture and terminal chamber (a size-normalized apertural length) was plotted against individual size in a Log-Log diagram (Figure 5A), and differences between the specimens located north and south of the Antarctic Subpolar Front were quantified through a two-group discriminant analysis. Alternatively, histograms and Gaussian kernel densities of the log-ratio between the size-normalized apertural length and the specimen major axis were plotted for both groups of specimens (Figure 5B). Resulting non-gaussian distributions were compared non-parametrically using Mann-Whitney U-test (H_0_: the two genotypes are taken from populations with equal median) and Kolmogorov-Smirnov D-test (H_0_: the two genotypes are taken from populations with equal distribution) [59]. All the computations were done using PAST v 2.00 [60].

## Supporting Information

Figure S1 ***SSU*** **rDNA based phylogenetic tree of** ***Globoconella inflata*** **.** Evolutionary relationships between 50 *SSU* rDNA clones of *G. inflata* from 13 localities in the Atlantic, Pacific and Indian Oceans (see Table 1 and Figure 1 for station names and locations). This Maximum Likelihood inference shows the relationships between the two phylotypes (Type I in red and Type II in blue). The bootstrap scores (500 replicates) greater than 80% are given next to branches. The scale and branch lengths are given in % of nucleotide substitution per site. The colors associated to leaf labels indicate geographic area of collection: blue = subpolar Indian Ocean; light blue = subpolar South Atlantic; Pink = South Atlantic north of the Subpolar Front; Yellow: Indian Ocean north of the Subpolar Front; red = North Atlantic; green = South Pacific; Orange = North Pacific. Circles associated to specific colors indicate clones sequenced from the same individuals.
(TIF)

Figure S2 **Principal components correlation coefficients.** PCA loading histograms, showing the correlations between the 13 depth-temperatures and the two first resulting Principal Components, representing 82.2% and 13.5% of the total variance, respectively. PC1 is a mean thermal state of the upper 500 m of the water column, whereas PC2 contrasts the temperatures of the first 125 m (negative weight) with the temperatures recorded between 150 and 500 m (positive weight) (weak contrast correspond to high PC2-value; high contrast correspond to low PC2-value).
(TIF)
