# Supplementary figures and images for: Worldwide Genotyping in the Planktonic Foraminifer Globoconella inflata: Implications for Life History and Paleoceanography
Source: PLoS One. 2011 Oct 20;6(10):e26665. doi: 10.1371/journal.pone.0026665 (PMC3197684; doi:10.1371/journal.pone.0026665)

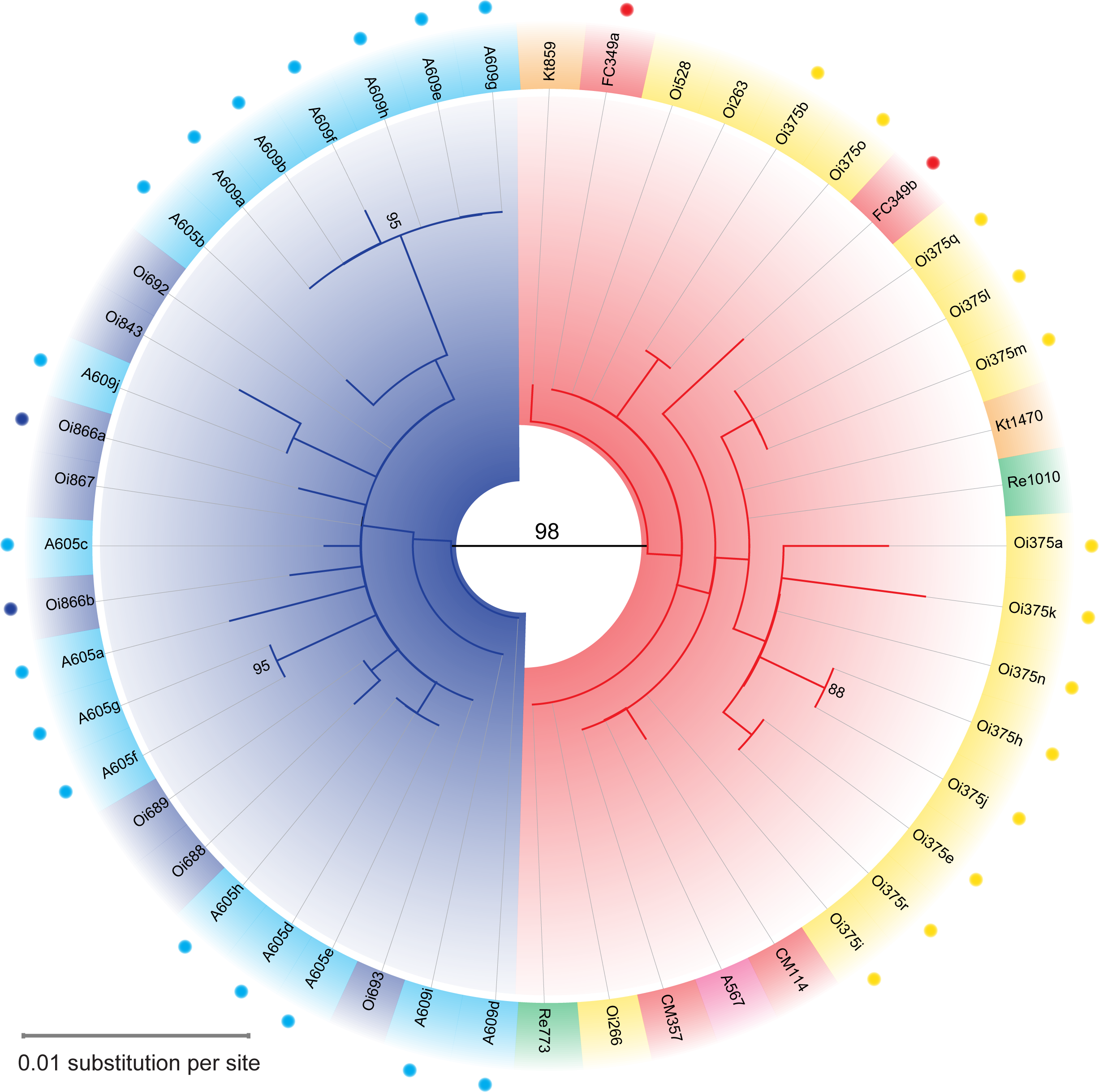

Supplement: Figure S1 — SSU rDNA based phylogenetic tree of Globoconella inflata . Evolutionary relationships between 50 SSU rDNA clones of G. inflata from 13 localities in the Atlantic, Pacific and Indian Oceans (see Table 1 and Figure 1 for station names and locations). This Maximum Likelihood inference shows the relationships between the two phylotypes (Type I in red and Type II in blue). The bootstrap scores (500 replicates) greater than 80% are given next to branches. The scale and branch lengths are given in % of nucleotide substitution per site. The colors associated to leaf labels indicate geographic area of collection: blue = subpolar Indian Ocean; light blue = subpolar South Atlantic; Pink = South Atlantic north of the Subpolar Front; Yellow: Indian Ocean north of the Subpolar Front; red = North Atlantic; green = South Pacific; Orange = North Pacific. Circles associated to specific colors indicate clones sequenced from the same individuals. (TIF) [file pone.0026665.s001.tif]

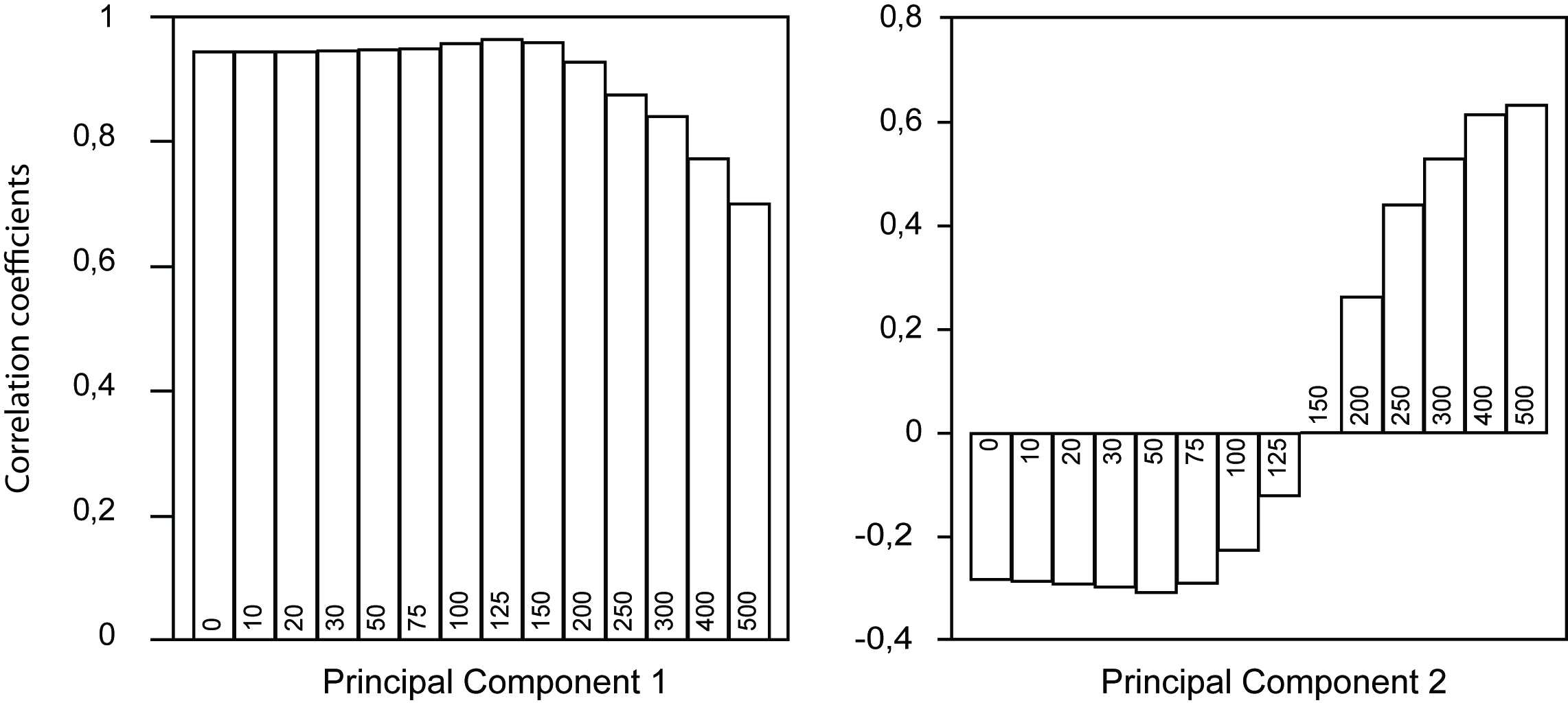

Supplement: Figure S2 — Principal components correlation coefficients. PCA loading histograms, showing the correlations between the 13 depth-temperatures and the two first resulting Principal Components, representing 82.2% and 13.5% of the total variance, respectively. PC1 is a mean thermal state of the upper 500 m of the water column, whereas PC2 contrasts the temperatures of the first 125 m (negative weight) with the temperatures recorded between 150 and 500 m (positive weight) (weak contrast correspond to high PC2-value; high contrast correspond to low PC2-value). (TIF) [file pone.0026665.s002.tif]
